# Does the primary screening test influence women’s anxiety and intention to screen for cervical cancer? A randomized survey of Norwegian women

**DOI:** 10.1186/1471-2458-14-360

**Published:** 2014-04-15

**Authors:** Emily A Burger, Mari Nygård, Dorte Gyrd-Hansen, Tron Anders Moger, Ivar Sonbo Kristiansen

**Affiliations:** 1 University of Oslo, Department of Health Management and Health Economics, PO BOX 1089, Blindern, Oslo 0137, Norway; 2 Cancer Registry of Norway, Oslo, Norway; 3 COHERE, Department of Business and Economics, Institute of Public Health, University of Southern Denmark, Odense, Denmark

**Keywords:** Mass screening, Human papillomavirus, Pap smear, Health policy

## Abstract

**Background:**

Countries must decide whether or not to replace primary cytology-based screening with primary human papillomavirus (HPV)-based screening. We aimed to assess how primary screening for an HPV infection, a sexually transmitted infection (STI), and the type of information included in the invitation letter, will affect screening intention.

**Methods:**

We randomized a representative sample of Norwegian women to one of three invitation letters: 1) Pap smear, 2) HPV testing or 3) HPV testing with additional information about the nature of the infection. Intention to participate, anxiety level and whether women intend to follow-up abnormal results were measured between groups using chi-squared and nonparametric Kruskal-Wallis tests. Determinants of intention were explored using logistic regression.

**Results:**

Responses from 3540 women were representative of the Norwegian population with respect to age, civil status and geographic location. No significant difference across invitation letters was found in women’s stated intention to participate (range: 91.8-92.3%), anxiety (39-42% were either quite or very worried) or to follow-up after an abnormal result (range: 97.1-97.6%). Strength of intention to participate was only marginally lower for HPV-based invitation letters, albeit significant (p-value = 0.008), when measured on a scale. Only 36–40% of respondents given the HPV invitations correctly understood that they likely had an STI.

**Conclusions:**

We found that switching to primary HPV screening, independent of additional information about HPV infections, is not likely to reduce screening participation rates or increase anxiety; however, women lacked the ability to interpret the meaning of an HPV-test result.

## Background

High participation and follow-up rates of population-based cervical cancer screening programs using cytology-based methods (Pap smear) have been effective in reducing the incidence and mortality of invasive cervical cancer in many developed countries [1]. Since 1995, the Norwegian Coordinated Cervical Cancer Screening Programme (NCCSP) has sent reminder letters to women aged 25–69 who have not had a registered Pap smear within the last three years and a second reminder is sent to women who have not had a registered smear within 12 months of the initial letter. The NCCSP also sends reminder letters to encourage women to follow-up with their physician in the case of an abnormal or unsatisfactory result. Currently, 80% of targeted women regularly participate in screening, while approximately 65-70% of women with a high-grade abnormal result return within one year [2].

Due to the causal link between cervical cancer and human papillomavirus (HPV), a common sexually transmitted infection (STI), diagnostic tests, which explicitly detect the presence of HPV, have been developed. Large, randomized controlled trials have shown that primary HPV testing can detect more high-grade precancerous lesions than conventional or liquid-based cytology (LBC) [3]. While treatment for HPV is currently unavailable, cellular changes associated with the infection can be monitored and treated to prevent invasive cervical cancer from developing. In response, several countries are considering replacing primary cytology-based screening with primary HPV-based screening. In Norway, an HPV-based algorithm for women aged ≥34 has been proposed [4] that involves primary HPV testing followed by reflex LBC (i.e., retesting the same sample) for women who test positive for high-risk HPV types. HPV testing may also facilitate less frequent screening [5] (e.g., extending the primary interval from 3 to 6 years has been suggested in Norway [4]).

Whether or not explicitly testing for an STI will affect screening adherence in Norway is not known. As in other countries, public understanding of HPV and its role in cervical cancer development is poor [6]. The most recent Norwegian survey [7] revealed that only one-third of women reported knowledge of HPV. Though few women may equate abnormal cytology results with sexual activity, there may be different psychosocial outcomes between women who test positive for detectable cellular or histological lesions and those who test positive for an asymptomatic HPV infection [8]. Some women may concentrate on the sexual transmissibility of HPV rather than its implications towards the development of cervical cancer. Fear, anxiety, distress and concern regarding sexual behavior have been reported as the prominent emotional reactions from testing positive for HPV [9-13]. Within relationships, questions about trust and blame may also arise [12]. In addition, women who link their positive HPV test to sexual activity may experience guilt, stigma and shame [14]; however, differences in psychosocial outcomes between HPV and Pap smear testing often converge within 6–12 months (implying that even if differences in anxiety are initially detected, they are unlikely to be long-lasting) [6,8]. The level of anxiety and stress experienced by the women may also be a function of understanding and how the results of a positive test are communicated [15].

One potential concern is that some specific subgroups may respond differently to primary HPV testing than others. In a qualitative study performed in the United States [16], women >55 years of age and married or women who were not currently sexually active were less likely to feel at risk of an infection with HPV. If primary HPV testing is implemented, and women older than 55 or in stable relationships equate their current self-perceived risk of having an STI with the need for HPV-based testing, they may not believe they require screening. On the other hand, in the Netherlands, a population-based randomized trial evaluated the efficacy of primary co-testing (i.e., primary cytology in combination with HPV testing) compared to cytology-based screening alone and reported no decrease in the attendance rate [17]. However, it is not clear whether these results can be extrapolated outside the context of a well-supported randomized trial (i.e., women may have been more motivated due to the possibility of more extensive surveillance offered in a trial setting) or for an HPV test that is not taken concomitantly with cytology, requiring women to rely solely on HPV-based screening.

Intention to perform an action is often cited as the most important predictor of behavioral performance or one’s true actions [18] and can be perceived as one’s motivation to perform. We aim to explore whether an invitation letter to a Pap smear screen (every three years) versus HPV-based screening (every six years) and the type of information included in the invitation letter influences women’s anxiety and intent to participate in screening and follow-up testing among Norwegian women. Secondly, we explored determinants of intention to participate in screening and investigated whether age- and civil status-specific subgroups of women, respond differently to explicitly testing for an STI compared to testing for abnormal cellular changes.

## Methods

### Study design

**Figure 1:**
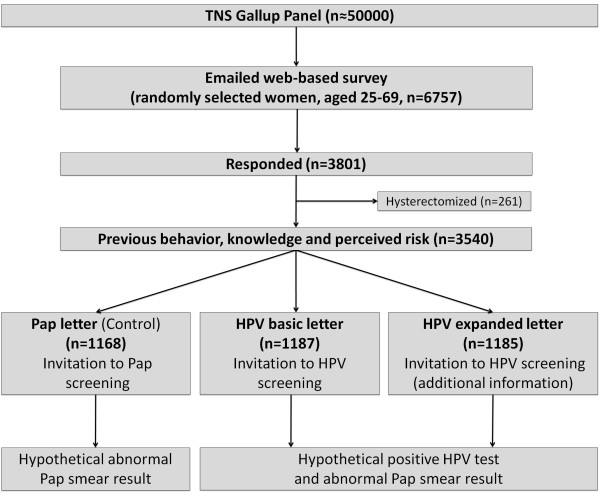
**Flow diagram.** Women were randomized to one of three invitation letters according to: 1) conventional practice using Pap smear-based screening (Pap letter), 2) to primary HPV testing with minimal information (HPV basic letter), or 3) to HPV testing with more explicit information about HPV infections (HPV expanded letter).

In 2011, we conducted a randomized web-based survey of a representative sample of Norwegian women using TNS Gallup’s active internet panel of more than 50,000 individuals. Baseline demographic characteristics, knowledge of cervical cancer and HPV infection, perceived risk of cervical cancer and anxiety towards developing STIs were obtained. To elicit the intent to participate in screening, women were randomized to one of three versions of cervical cancer screening invitation letters (Figure 1). The first version of the invitation (herein referred to as the “Pap letter”) used verbatim text from the current reminder letter sent to women from the NCCSP recommending triennial Pap smear screening [2]. The second version (herein referred to as the “HPV basic letter”) preserved the wording of the Pap letter but stated that HPV testing would replace Pap smear testing and informed women that they would only need to be screened every six years (to reflect the current proposal for primary HPV testing in Norway [4]). The letter also informed women that in the event of a positive HPV test, their same sample would be retested for cellular changes (i.e., reflex LBC). The third invitation letter (herein referred to as the “HPV expanded letter”) was identical to the HPV basic letter, but included additional information regarding the ease of transmission of HPV as well as stating that it is possible to have the infection for many years without knowing it. This letter targeted women who may not believe they are at risk of HPV due to age or current sexual behavior. All three letters stated that cervical cancer is caused by an infection with HPV and is transmitted sexually (See Additional file 1 for translated invitation letters). Lastly, women were asked to read a letter, based on actual text from the NCCSP, which provided information about a hypothetical positive test result. We developed the survey using a multi-stage, iterative process. We formulated the vignettes and questions to evaluate our objectives (in collaboration with stakeholders from the Cancer Registry of Norway), using familiar and neutral language, and included response categories that were mutually exclusive and collectively exhaustive. At an early stage of survey development, a convenience sample of women (n = 55) provided feedback on question understanding, interpretation and relevance. Finally, a link to the updated electronic version of the survey was emailed to a small sample of women from the TNS Gallup panel, in which 107 provided feedback on the technical aspects of survey administration, as well as an opportunity to provide general open-ended comments.

In keeping with the proposed primary HPV testing algorithm [4], women randomized to the HPV basic or expanded letter were asked to imagine they were HPV-positive and that there was evidence of abnormal cellular changes, while women who received the Pap letter were only told to imagine they had abnormal cellular changes. Questions explored willingness to participate in screening and follow-up testing, level of understanding and anxiety associated with the positive test letter. Intention was captured by binary response (yes/no), the strength of intent to participate was elicited on a 10-point scale (1: definitely not attend; 10: definitely attend) and anxiety was measured on a 5-point scale (1: not worried at all; 5: very worried). Informed consent was obtained from all study participants and de-identified for analysis. The study design and materials were reviewed and approved by the regional ethical committee.

### Participants

Following pilot work, a power calculation for a two-sample comparison of proportions determined that 1075 respondents in each of the three randomized groups (n = 3225 in total) would be needed for 80% power with a significance level of 0.05, to detect a 3.5% difference (experts deemed clinically important) between expressed participation rates. Based on published estimates of the prevalence of hysterectomized women in Norway [19], we expected to exclude 5% of respondents aged 40–50 and 10% of respondents aged 50–60. The survey was sent out to 6757 women, a random sample of women aged 25–69 in the panel. A target response of 3801 women (56%) was obtained within two weeks, after which the website was inactivated. The response rate may be underestimated, as some women who may have been willing to respond did not have the opportunity to do so after the required response level was achieved. After removal of hysterectomized women (6.9%), the survey sample consisted of 3540 women.

### Data analysis

We calculated proportions for binary responses and means for the scale responses. We conducted Chi-squared tests of statistical significance for the difference between proportions and nonparametric analysis of variance (Kruskal-Wallis) to test the differences between groups for questions measured on a continuous scale. Odds ratios (ORs) were derived from univariable and multivariable logistic regression models for the binary dependent variable of intent to participate. The dependent variable was set to equal 1 if the respondent intended to participate, and to zero if they did not intend to participate. Independent variables were selected to reflect author hypotheses and factors, which have been associated with screening participation in previous studies. In addition to the main effects, we studied whether invitation letter type interacts with age (25–39, 40–59, 60–69) and civil status (single vs. cohabiting/married) with regard to participation rate. The α-level was set at 0.05. All data was analyzed in STATA, release 12 (StataCorp, College Station, TX).

## Results

The mean age of the final sample of 3540 women was 45.1 years. The baseline demographic characteristics were evenly distributed among the randomized groups (Table 1) and generally representative of the Norwegian population for age, geographic location, level of income and civil status; however, the sample was slightly over-represented by higher educated women.

**Table 1:** Background characteristics of women randomized to one of three invitation letters to participate in cervical cancer screening

**Variables**	**Survey randomization**				
	**Pap letter (n = 1 168)**	**HPV basic letter (n = 1 187)**	**HPV expanded letter (n = 1 185)**	**Total (n = 3 540)**	Norwegian population*
**Age** (years), mean (SD)	45.3 (12.3)	45.3 (11.9)	44.7 (12.1)	45.1 (12.1)	
**Age distribution** (years)					
<30	147 (12.6)	126 (10.6)	155 (13.1)	428 (12.1)	11.1%
30-39	274 (23.5)	295 (24.9)	288 (24.3)	857 (24.2)	23.7%
40-49	278 (23.8)	308 (26.0)	301 (25.4)	887 (25.1)	24.8%
50-59	286 (24.5)	287 (24.2)	272 (23.0)	845 (23.9)	21.8%
60-69	183 (15.7)	171 (14.4)	169 (14.3)	523 (14.8)	18.6%
**Personal income** (Kroner)	n = 1 079	n = 1 094	n = 1 108	n = 3 281	
<200 000	134 (12.4)	127 (11.6)	135 (12.2)	396 (12.1)	22.5%
200 000–399 999	521 (48.3)	541 (49.5)	524 (47.3)	1 586 (48.3)	48.0%
400 000–599 999	371 (34.4)	361 (33.0)	373 (33.7)	1 105 (33.7)	20.8%
600 000–799 999	37 (3.4)	47 (4.3)	69 (6.2)	153 (4.7)	6.2%
≥800 000	16 (1.5)	18 (1.6)	7 (0.6)	41 (1.2)	2.5%
**Geographic location**					
Oslo	293 (25.1)	325 (27.4)	284 (24.0)	902 (25.5)	27.8%
South East (excluding Oslo)	298 (25.5)	312 (26.3)	306 (25.8)	916 (25.9)	25.1%
South West	372 (31.9)	350 (29.5)	397 (33.5)	1 119 (31.6)	30.9%
North	205 (17.6)	200 (16.5)	198 (16.7)	603 (17.0)	16.2%
**Civil status**	n = 1 104	n = 1 109	n = 1 108	n = 3 321	
Single	319 (27.3)	283 (25.5)	311 (28.1)	913 (27.5)	28.7%
Married/Cohabiting	785 (67.2)	826 (74.5)	797 (71.9)	2408 (72.5)	71.3%
**Education**	n = 1 168	n = 1 186	n = 1 184	n = 3 538	
≤High school or vocational school	443 (37.9)	433 (36.5)	448 (37.8)	1 324 (37.4)	61.0%
≥Bachelor degree	725 (62.1)	753 (63.5)	736 (62.2)	2 214 (62.6)	39.0%
**Born in Norway**	n = 1 165	n = 1 183	n = 1 183	n = 3 531	
Yes	1 106 (94.9)	1 127 (95.3)	1 111 (93.9)	3 344 (94.7)	n.a.

Values are numbers (percentages) unless otherwise stated.

CC, cervical cancer; HPV, human papillomavirus; n.a., not available; (NOK8.004 = €1).

*Statistics Norway (http://www.ssb.no/english).

Questions eliciting general knowledge and anxiety (Table 2) indicated that 42% of the respondents identified the primary cause of cervical cancer as a virus and approximately half (53%) of the women reported having previously heard of HPV. Of those women who had heard of HPV, 82% identified HPV as an STI. Nearly all women (93%) felt that Pap smears were either quite important or very important for the prevention of cervical cancer and 94% reported having had at least one previous Pap smear. The majority of women (63%) stated they were not very worried about developing cervical cancer, and 88% stated that they were not worried about contracting an STI. The proportion of women who were least anxious towards contracting an STI peaked among the oldest age group (95%) as well as among women in committed relationships (93%). We found no significant difference in women’s intent to participate in screening (Pap letter: 92.3%, HPV basic letter: 91.8%, HPV expanded letter: 92.2%) or follow-up (Pap letter: 97.6%, HPV basic letter: 97.1%, HPV expanded letter: 97.2%) between the invitation letters when captured by binary response (Table 3). When the strength of intention was elicited on a scale, the mean intent to participate in screening (Pap letter: 8.40, HPV basic letter: 8.29, HPV expanded letter: 8.24) and follow-up (Pap letter: 9.34, HPV basic letter: 9.14, HPV expanded letter: 9.16) were marginally lower for those women who received the HPV invitations, albeit significant (p-value = 0.008 and p-value = 0.002, respectively). Though anxiety linked to an abnormal result was present (approximately 39-42% were either quite or very worried), women did not worry significantly more or less between the invitation letters (p-value = 0.184). However, the ability to correctly interpret a positive result was lacking; only 37% of women randomized to the HPV basic letter and 40% of women randomized to the HPV expanded letter understood their positive HPV test indicated they had an STI. Women were equally as likely to reveal their abnormal Pap smear or HPV-positive result to their partner; however, up to 5% fewer women who received the positive HPV result, in addition to abnormal cellular changes, stated they would tell a close friend (p-value = 0.01), compared to the women who were given only an abnormal Pap smear result. After the inclusion of relevant covariates, logistic regression confirmed that the odds of participating were not statistically different after receiving different versions of the invitation letter (Table 4).

**Table 2:** Responses for questions eliciting knowledge and anxiety by randomized group

Variables*	**Survey randomization**			
	**Pap letter (n = 1 168)**	**HPV basic letter (n = 1 187)**	**HPV expanded letter (n = 1 185)**	**Total (n = 3540)**
**Perceived health**				
Neither good nor bad	156 (13.4)	151 (12.7)	149 (12.6)	456 (12.9)
Bad/Very bad	52 (4.5)	44 (3.7)	34 (2.9)	130 (3.7)
**Have had previous Pap smear**				
Yes	1 092 (93.8)	1 125 (95.1)	1 112 (94.2)	3 329 (94.3)
**Have had previous dysplasia**				
Yes	245 (21.0)	266 (22.5)	257 (21.7)	768 (21.8)
Don't know	60 (5.2)	48 (4.1)	63 (5.3)	171 (4.8)
**Primary cause of CC?**				
Genetics	245 (21.0)	254 (21.4)	244 (20.6)	743 (21.0)
A virus	514 (44.0)	477 (40.3)	493 (41.6)	1 484 (42.0)
Hormones	120 (10.3)	125 (10.6)	133 (11.2)	378 (10.7)
Smoking	9 (0.8)	12 (1.0)	10 (0.84)	31 (0.9)
Other	25 (2.1)	33 (2.8)	23 (1.94)	81 (2.3)
Don't know	254 (21.8)	284 (24.0)	281 (23.7)	819 (23.2)
**Heard about HPV?**				
Yes	627 (53.8)	628 (53.0)	608 (51.4)	1 863 (52.7)
**Believes HPV is transmitted through**^**a**^				
Air	0 (0.00)	4 (0.6)	4 (0.7)	8 (0.4)
Drinking water	1 (0.2)	1 (0.2)	2 (0.3)	4 (0.2)
Food	1 (0.2)	1 (0.2)	3 (0.5)	5 (0.3)
Sexual contact	526 (83.9)	505 (80.4)	496 (81.6)	1 527 (82.0)
Don't know	99 (15.8)	117 (18.6)	103 (16.9)	319 (17.1)
**Worried about developing CC?**				
Very little or not at all	734 (62.9)	746 (63.0)	738 (62.4)	2 218 (62.7)
Some	372 (31.9)	378 (31.9)	369 (31.2)	1 119 (31.7)
Quite worried or very worried	61 (5.2)	61 (5.1)	76 (6.4)	198 (5.6)
**Worried about developing STI?**				
Very little or not at all	1 032 (88.4)	1 046 (88.2)	1 050 (88.7)	3 128 (88.4)
Some	90 (7.7)	92 (7.8)	81 (6.8)	263 (7.4)
Quite worried or very worried	46 (3.9)	48 (4.1)	53 (4.5)	147 (4.2)
**Importance of Pap smear to prevent CC?**				
Very little or not at all	15 (1.3)	21 (1.8)	20 (1.7)	56 (1.6)
Some	59 (5.1)	65 (5.5)	68 (5.7)	192 (5.4)
Quite important or very important	1 094 (93.7)	1 101 (92.8)	1 097 (92.6)	3 292 (93.0)

Values are numbers (percentages) unless otherwise stated.

CC, cervical cancer; HPV, human papillomavirus; STI, sexually transmitted infection.

^*^A maximum of 13 women (0.4%) chose not to answer on any given question.

^a^Answering the question was contingent on having previously heard of HPV.

**Table 3:** Primary results of intention to screen and follow-up abnormal results, anxiety and willingness to disclose abnormal result, by randomized group

Variables*	**Survey randomization**			**p-value**
	**Pap letter (n = 1 168)**	**HPV basic letter (n = 1 187)**	**HPV expanded letter (n = 1 185)**	
**Intend to participate in CC screening?**				
Yes	92.3 (90.8-93.8)	91.8 (90.3-93.4)	92.2 (90.7-93.7)	0.906
**How likely to participate? (scale 1–10)**^**a**^				
Mean (CI)	8.40 (8.27-8.54)	8.29 (8.15-8.41)	8.24 (8.10-8.36)	0.008^b^
**Intend to participate in follow-up control?**				
Yes	97.6 (96.7-98.5)	97.1 (96.2-98.1)	97.2 (96.3-98.2)	0.751
**How likely to participate in follow-up? (scale 1–10)**^**a**^				
Mean (CI)	9.34 (9.24-9.44)	9.14 (9.04-9.24)	9.16 (9.04-9.25)	0.002^c^
**Worried about abnormal test result?**				0.184
Not at all or very little	14.1 (12.1-16.1)	12.9 (11.0-14.8)	12.2 (10.3-14.0)	
Some	46.9 (44.0-49.7)	45.3 (42.4-48.1)	45.9 (43.0-48.7)	
Quite or very worried	39.0 (36.2-41.8)	41.8 (39.0-44.6)	42.0 (39.2-44.8)	
**Interpretation of positive test?**				<0.001
Unlikely/very unlikely I have an STI	70.4 (67.8-73.1)	63.5 (60.7-66.2)	60.4 (57.7-63.2)	
Likely or very likely I have an STI	29.6 (27.0-32.2)	36.5 (33.8-39.3)	39.6 (36.8-42.4)	
**Would tell partner about test result?**				0.913
Yes	91.8 (90.2-93.4)	92.1 (90.5-93.6)	92.4 (90.9-93.9)	
Don't know	7.2 (5.7-8.7)	6.8 (5.3-8.2)	6.33 (5.0-7.7)	
**Would tell close friends about test result?**				0.010
Yes	30.5 (27.9-33.2)	28.5 (25.9-31.1)	25.7 (23.2-28.2)	
Don't know	39.5 (36.7-42.3)	36.2 (33.5-39.0)	39.7 (36.9-42.5)	

Values are percentages (CI) unless otherwise stated.

CC, cervical cancer; CI, confidence interval; STI, sexually transmitted infection.

^*^A maximum of 19 women (0.5%) chose not to answer on any given question.

^a^CI for nonparametric distribution calculated by bootstrapping and using bias-corrected estimates.

^b^Letter 1 vs Letter 2: P-value = 0.05, Letter 1 vs Letter 3: P-value = 0.002.

^c^Letter 1 vs Letter 2: P-value = 0.002, Letter 1 vs Letter 3: P-value = 0.001.

**Table 4:** Results of the univariable and multivariable logistic regression assessing factors associated with women who are likely to participate in screening

	**Univariable model**			Multivariable model*		
	**OR**	**95% CI**	**p-value**	**OR**	**95% CI**	**p-value**
**Letter type**			0.91			0.64
Pap letter	1	--		1	--	
HPV basic letter	0.94	(0.69-1.27)	0.68	0.87	(0.61-1.24)	0.46
HPV expanded letter	0.99	(0.73-1.34)	0.934	1.01	(0.70-1.46)	0.92
**Self perceived health**			0.001			0.08
Good or very good	1	--		1	--	
Neither good nor bad	0.80	(0.57-1.14)	0.22	0.97	(0.62-1.52)	0.91
Bad or very bad	0.41	(0.25-0.67)	<0.001	0.49	(0.27-0.91)	0.02
**Previous Pap smear**						
No/don't know	1	--		1	--	
Yes	4.60	(3.24-6.53)	<0.001	3.09	(1.92-4.95)	<0.001
**Primary cause of CC?**						
Doesn't know, (stated other than virus)	1	--		1	--	
Virus	1.47	(1.13-1.90)	0.004	1.34	(0.97-1.84)	0.08
**Worried about developing CC?**			<0.001			0.001
Very little or not at all	1	--		1	--	
Some	2.03	(1.50-2.75)	<0.001	1.92	(1.32-2.80)	0.001
Quite worried or very worried	2.53	(1.23-5.20)	0.01	2.65	(0.92-7.63)	0.07
**Worried about developing an STI?**			0.78			0.72
Very little or not at all	1	--		1	--	
Some	1.18	(0.72-1.95)	0.50	0.96	(0.51-1.83)	0.91
Quite worried or very worried	1.08	(0.57-2.02)	0.82	0.71	(0.31-1.63)	0.42
**Perceived risk of CC compared to others?**			<0.001			0.04
Lower	1	--		1	--	
Same	1.67	(1.27-2.20)	<0.001	1.47	(1.05-2.06)	0.03
Higher	3.64	(1.86-7.14)	<0.001	2.13	(0.96-4.72)	0.06
**Importance of Pap smear to prevent CC?**			<0.001			<0.001
Very little or not at all	1	--		1	--	
Some	1.01	(0.52-1.96)	0.97	0.75	(0.33-1.73)	0.51
Quite important or very important	5.89	(3.24-10.69)	<0.001	3.50	(1.64-7.50)	0.001
**Interpret the results from a positive test?**						
Unlikely/Very unlikely STI	1	--		1	--	
Likely/Very likely STI	2.13	(1.59-2.87)	<0.001	1.70	(1.17-2.45)	0.005
**Would tell partner about test result?**			<0.001			<0.001
Yes	1	--		1	--	
No	0.15	(0.08-0.29)	<0.001	0.21	(0.09-0.48)	<0.001
Don't know	0.59	(0.39-0.89)	0.01	0.74	(0.44-1.23)	0.25
**Would tell close friends about test result?**			<0.001			0.15
Yes	1	--		1	--	
No	0.60	(0.44-0.82)	0.001	0.75	(0.52-1.09)	0.14
Don't know	0.96	(0.69-1.33)	0.79	1.02	(0.69-1.50)	0.92

CC, cervical cancer; CI, confidence interval; OR, odds ratio; STI, sexually transmitted infection.

*In addition to variables in the table, odds ratios (ORs) were adjusted for age, income, education, and civil status using listwise deletion of missing observations. Model fit was good, indicated by a non-significant Hosmer-Lemeshow test (p-value = 0.15).

Socio-demographic factors were significantly associated with intention to participate in the univariable models, though effects did not remain significant in the multivariable model (see Additional file 1 Appendix Table). Women were more likely to state an intention to participate 1) stated that they had received a previous Pap smear (OR 3.09, 95% CI 1.92-4.95), 2) were to some extent worried about developing cervical cancer (OR 1.92, 95% CI 1.32-2.80), 3) considered Pap smear screening very important to prevent cervical cancer (OR 3.50, 95% CI 1.64-7.50), or 4) were able to link the positive test result with an STI (OR 1.70, 95% CI 1.17-2.45). Women who felt they were in bad or very bad self-perceived health (OR 0.49, 95% CI 0.27-0.91) or would not tell a partner about the results (OR 0.21, 95% CI 0.09-0.48) had a significantly reduced odds of intent to participate. When we tested for interaction terms between the letters and age, and the letters and civil status in the univariable logistic model, there were no significant effects (χ^2^ = 2.55, df = 4, p-value = 0.64 and χ^2^ = 1.02, df = 2, p-value = 0.60, respectively) and thus, the variables were not included in the models presented in Table 4.

## Discussion

The results of this randomized survey suggest that neither a switch from Pap smear to HPV testing nor more explicit information about the infectious nature of the virus are likely to increase anxiety or reduce screening attendance. We cannot exclude the impact of HPV testing on the strength of intent to participate as measured on a scale; however, the effect is small and not likely to be clinically relevant. Rather, our results from the multivariable regression model indicate that factors such as: confidence in the benefits of screening, anxiety towards developing cervical cancer, perceived risk and ability to correctly interpret results have a greater impact on intention to screen, beyond what changing the screening test might induce.

Our results suggest that the ability to associate an HPV infection as a sexually transmitted infection was lacking, and the indifference to screening test method may be influenced by this high level of unawareness with HPV. Nearly half of the women had never heard of HPV, and the majority of women presented with the HPV basic and expanded letter versions misinterpreted the sexually transmitted nature of the infection, regardless of being explicitly told in the invitation letter. Women who received the Pap letter were also told in the letter that most cellular changes were caused by an infection with HPV—and were even less likely to state a connection between cellular abnormalities and the STI. Women may be less able to accurately weigh the benefits and harms of screening due to a lack of understanding. In our sample, we found that the ability to correctly interpret an HPV-test result was positively associated with intention to participate, even after adjusting for education. However, the effect of a more informed population on participation would still be uncertain, as the relationship is not necessarily causal. The women in our study did not seem to be more anxious towards receiving a positive HPV result even though testing positive for an STI has been shown to be stressful and evoke stigma. Even if present, studies have shown that over time, HPV-positive related anxiety does not inflict additional burden beyond what having an abnormal Pap smear imposes [6,20]. It should be noted that regardless of test method, an overwhelming proportion of respondents reported anxiety towards an abnormal test result. Additional analyses that address the appropriate communication of results could help alleviate unnecessary anxiety for women faced with an abnormal test result. As expected, anxiety towards developing an STI was inversely related to age and was lower among women in committed relationships; however, this anxiety did not affect overall intention to participate. On the other hand, women reporting anxiety towards developing cervical cancer were more likely to report intention to participate. We also found that women were slightly more hesitant to reveal a positive HPV result to their close friends compared to a Pap smear result (Table 3); yet, whether this suggests there is more stigma associated with the HPV result is unclear as measuring willingness to tell friends is a sub-optimal proxy for stigma. Regardless of the effect on attendance, it is clear that educational campaigns should accompany changes in screening programs to ensure women are fully informed, understand what they are being screened for and how to interpret their results.

Specific type of cervical cancer screening test may not be one of the motivating (or de-motivating) factors that influences participation. One possible explanation would be if the rationale to attend screening in Norway stems from a sense of duty. This "obligation factor” has been tied to screening previously, and is often documented in welfare countries where state-run health care systems dominate [21]. In neighboring Sweden participation rates in cervical cancer screening programs are similarly high (over 90% participate at least once), but a reported one-third of women were unaware of which type of cancer they were being screened for [22]. Similarly, publicly provided screening programs have been documented to increase the perceived value of the screening program when compared to programs offered through a private healthcare provider, where individuals may comply due to the fact they trust the source of the recommendation [23]. In Norway, where screening programs are almost exclusively publicly funded, qualitative research evaluating the decision-making process surrounding screening uptake revealed that some women likened the welfare state’s healthcare system to a “mother” figure [24]. In addition, many women may simply attend in order to have the personal reassurance they are disease free. Any of the above reasons could provide the basis that current participation rates could likely remain high irrespective of future screening test method.

Previous knowledge of HPV was higher than what has been previously reported in 2005 [7], though an increase in awareness of HPV is expected given the media attention surrounding the introduction of the HPV vaccine in Norway in 2009. Our sample’s self-reported history of having had at least one previous Pap smear was in line with age-specific rates reported in a recent survey and verified using registry data [25]. Participation rates in Norway have been shown to be influenced by civil status [26], not affected by educational status and decrease with age [2,26]. We found similar trends for intention to participate in the univariable model, though the effects were no longer significant in the multivariable model, similar to the other study [26]. The associations we obtained between knowledge, perceived risk, previous behavior and anxiety with participation have previously been documented [27] and our primary finding of no difference in willingness to participate between primary cytology and HPV testing are similar to those found in the POBASCAM [17] randomized controlled trial, where in fact, actual participation rates in the trial increased compared to historic levels.

### Limitations

Confounders and statistical variation between letter versions are likely minimized due to randomization and our large sample size; however, generalizing our results to the Norwegian population may be mitigated by 1) not allowing all women adequate time to respond to the survey (due to the target number of women reached within two weeks) and 2) the over representativeness of women with a university education when compared to the Norwegian population (Table 1). These factors may introduce selection bias, particularly if non-responders are more concerned with primary HPV-based screening than responders. Nevertheless, the overall response rate was greater than 50%, and intended participation was not affected when we adjusted for educational attainment, adding strength to the ability to generalize our study to the Norwegian population. As expected, actual participation rates recorded by the Cancer Registry of Norway [2] do not reach the same magnitude as stated intention measured in our survey. It is a well-known maxim that what people say is not what they actually do; therefore, our results are subject to hypothetical bias due to not measuring actual behavior. In addition, capturing responses to a hypothetical positive test result may reduce the ability to measure anxiety, as women may not internalize their result. Similarly, we cannot discount that women may have only scanned the invitation letter without noticing the switch in test method for those with the HPV invitation letters. Consequently, the null effect of HPV testing on participation may be due to the lack of careful reading and whether there is an actual corollary of introducing the new technology is not certain given that we measured intention rather than action. In order to minimize survey fatigue, we did not provide additional material to aid interpretation of a positive HPV-result, but as any organized screening program switches to primary HPV screening, it is likely more in-depth material will accompany any positive result and could impact understanding.

### Policy implications

It is important for policy-makers to gauge the impact primary HPV testing will have on attendance prior to implementation in Norway. Our findings are similar to results from a previous study conducted within a large randomized trial setting for primary screening [17] and to a contingent value study conducted in the UK for HPV testing in a triage setting [28]. The impact of introducing HPV as a primary screening test on participation is small if at all present and strengthens the argument for its implementation in Norway, as evidence continues to grow suggesting that HPV testing will likely not induce prolonged adverse psychological effects. Larger health benefits in combination with less frequent screening intervals have been shown to be less costly to society than the current Pap smear-based program in Norway [29]. In addition, as half of the cervical cancers in Norway are among those who choose not to regularly participate in screening, attendance in the program has the potential to increase if self-sampling kits are sent to non-attenders [30], an opportunity that is not as readily available with Pap smear-based screening.

## Conclusion

Results indicate that, in Norway, switching to primary HPV screening, independent of additional information on HPV infections, is neither likely to reduce screening participation rates nor increase anxiety. However, women lacked the proper interpretation of their HPV-test result and it is uncertain whether increased awareness of HPV and its implications will impact future participation rates in primary HPV screening programs.

## Competing interests

EAB, DGH and TAM have no interests to declare. MN and ISK are members of the Norwegian Directorate of Health’s national advisory board for cervical cancer screening.

## Author contributions

EAB, MN, DGH and ISK designed the study. EAB and TAM analyzed the data. EAB, MN, DGH, ISK interpreted results. EAB drafted the manuscript and all authors aided revision and accepted the final version.

## Pre-publication history

The pre-publication history for this paper can be accessed here:

http://www.biomedcentral.com/1471-2458/14/360/prepub

## Supplementary Material

Additional file 1 Translated survey invitations letters and additional results. Click here for file
