# Supplementary material for: Does the primary screening test influence women’s anxiety and intention to screen for cervical cancer? A randomized survey of Norwegian women
Source: BMC Public Health. 2014 Apr 15;14:360. doi: 10.1186/1471-2458-14-360 (PMC4021156; doi:10.1186/1471-2458-14-360)
Supplement: Additional file 1 — Translated survey invitations letters and additional results. [file 1471-2458-14-360-S1.doc]

**Supplemental Appendix**

**Accompanying the manuscript:**

Pap-smear or HPV-test: Does it influence women’s anxiety and intention to screen for cervical cancer? A randomized survey of Norwegian women

Emily A. Burger, MPhil

Mari Nygård, MD PhD

Dorte Gyrd-Hansen, PhD

Tron Moger, PhD

Ivar Sonbo Kristiansen, MD, MPH, PhD

**Invitation letters:** The three invitation letters translated from the original Norwegian-language version. Red text indicates changes from the Pap letter. Blue text indicates changes from HPV basic letter.

**Pap letter.**

**<<<**Pap smears taken from the cervix can save lives. In Norway, health authorities and the Population-based Screening Program recommend that all women between the ages of 25 to 69 receive a Pap smear every three years. Pap smears can detect serious cellular changes that can develop into cervical cancer. There is effective treatment for serious cellular changes. Cervical cancer is caused by an infection with human papillomavirus (HPV). HPV can occur in both men and women, and is transmitted sexually. Most HPV-infections are not dangerous and go away on their own. In some cases, the infection does not go away, but cause serious cellular changes that can develop into cervical cancer. Treatment of serious cellular changes reduces the risk of cervical cancer. Over half of the women that get cervical cancer have not taken a gynecological exam. Make an appointment at your doctor’s, attend, ask for information, and take the test.**>>>**

**HPV basic letter.**

**<<<**Pap smears taken from the cervix can save lives. In Norway, health authorities and the Population-based Screening Program recommend that all women between the ages of 25 to 69 are tested for human papillomavirus (HPV) every six years. Cervical cancer is caused by an infection with human papillomavirus (HPV). Testing for HPV is more likely to detect women that are more likely to develop cervical cancer. HPV can occur in both men and women, and is transmitted sexually. Most HPV-infections are not dangerous and go away on their own. In some cases, the infection does not go away, but cause serious cellular changes that can develop into cervical cancer. Treatment of serious cellular changes reduces the risk of cervical cancer. HPV-testing is replacing Pap smear as the primary test for cervical cancer and is performed in the same manner. Pap smears are taken every three years. If the HPV-test determines that you do not have an HPV-infection, then you do not need to take a new test for six years. If an HPV-infection is detected, a Pap smear will also be conducted on the same sample and you will receive both test results. Over half of the women that get cervical cancer have not taken a gynecological exam. Make an appointment at your doctor’s, attend, ask for information, and take the test. **>>>**

**HPV expanded letter.**

**<<<**Pap smears taken from the cervix can save lives. In Norway, health authorities and the Population-based Screening Program recommend that all women between the ages of 25 to 69 are tested for human papillomavirus (HPV) every six years. Cervical cancer is caused by an infection with human papillomavirus (HPV). Testing for HPV is more likely to detect women that are more likely to develop cervical cancer. HPV can occur in both men and women, is transmitted sexually between both men and women, and between partners of the same sex. Women with many sexual partners as well as women with just one partner can get HPV if the partner has been in contact with the virus. Because the infection does not give symptoms, it’s possible to have the infection without knowing it. Most HPV-infections are not dangerous and go away on their own. In some cases, the infection does not go away, but cause serious cellular changes that can develop into cervical cancer. Treatment of serious cellular changes reduces the risk of cervical cancer. HPV-testing is replacing Pap smear as the primary test for cervical cancer and is taken in the same way. Pap smears are taken every three years. If the HPV-test determines that you do not have an HPV-infection, then you do not need to take a new test for six years. If an HPV-infection is detected, a Pap smear will also be conducted on the same sample and you will receive both test results. Over half of the women that get cervical cancer have not taken a gynecological exam. Make an appointment at your doctor’s, attend, ask for information, and take the test. **>>>**

**Result letters:** The two letters informing women about their positive results translated from the original Norwegian-language version. Red text indicates changes from the Pap result letter.

**Results for respondents receiving the Pap letter.**

**<<<**Information letter regarding the need for a control of your Pap smear.

In Norway, all Pap smears are registered by the Cancer Registry if you have not opted out. Your sample has been registered and it is recommended that you return for a control. Such controls are recommended when irregular cells are detected and are uncertain. It is important that you clarify the need for a control with your own doctor. Additional information, including frequently asked questions and answers can be found at www.kreftregisteret.no/livmorhals. **>>>**

**Results for respondents receiving either the HPV basic or HPV expanded letters.**

**<<<**Information letter regarding the need for a control of your HPV-test.

In Norway, all human papillomavirus (HPV) tests are registered by the Cancer Registry if you have not opted out. Your test for HPV has been registered and it is recommended that you return for a control. Such controls are recommended when an HPV-infection and irregular cells are detected. It is important that you clarify the need for a control with your own doctor. Additional information, including frequently asked questions and answers can be found at www.kreftregisteret.no/livmorhals. **>>>**

| **Appendix Table 1.** Results from the univariable and multivariable logistic regression for the demographic variables which were not shown on Table 4 of the main manuscript. | | | | | | | | | |
| --- | --- | --- | --- | --- | --- | --- | --- | --- | --- |
|  |  | **Univariable Model** | | |  | **Multivariable Model*** | | |  |
|  |  | **OR** | **95% CI** | **p-value** |  | **OR** | **95% CI** | **p-value** |  |
|  | Age |  |  | 0.05 |  |  |  | 0.55 |  |
|  | 25-39 | 1 | -- |  |  | 1 | -- |  |  |
|  | 40-59 | 1.07 | (0.82-1.41) | 0.611 |  | 1.03 | (0.72-1.48) | 0.85 |  |
|  | 60-69 | 0.71 | (0.50-1.00) | 0.05 |  | 0.83 | (0.54-1.30) | 0.42 |  |
|  | Household income |  |  | <0.001 |  |  |  | 0.95 |  |
|  | Under 399 999 | 1 | -- |  |  | 1 | -- |  |  |
|  | 400 000-599 999 | 1.32 | (0.91-1.92) | 0.14 |  | 1.08 | (0.69-1.69) | 0.74 |  |
|  | 600 000-799 999 | 1.79 | (1.21-2.65) | 0.004 |  | 1.01 | (0.59-1.71) | 0.98 |  |
|  | 800 000-999 999 | 2.23 | (1.47-3.40) | <0.001 |  | 1.24 | (0.69-2.22) | 0.48 |  |
|  | 1 000 000 or more | 1.98 | (1.28-3.05) | 0.002 |  | 1.12 | (0.62-2.04) | 0.7 |  |
|  | Education |  |  |  |  |  |  |  |  |
|  | ≤High school or vocational school | 1 | -- |  |  | 1 | -- |  |  |
|  | ≥Bachelor degree | 1.26 | (0.98-1.62) | 0.07 |  | 1.23 | (0.89-1.69) | 0.22 |  |
|  | Civil status |  |  |  |  |  |  |  |  |
|  | Married/Cohabiting | 1 | -- |  |  | 1 | -- |  |  |
|  | Single | 0.57 | (0.44-0.74) | <0.001 |  | 0.72 | (0.48-1.09) | 0.13 |  |

CC, cervical cancer; CI, confidence interval; OR, odds ratio; STI, sexually transmitted infection
